# Rush to Judgment

**DOI:** 10.1371/journal.pmed.0030071

**Published:** 2006-01-31

**Authors:** Richard Winkel

**Affiliations:** **1**University of MissouriColumbia, MissouriUnited States of America

How have Auvert et al. [ 1] controlled for the nonrandomization implicit in using a pool of men who want to be circumcised? Such self-selection increases the likelihood of recruiting men who are experiencing sexual difficulties—such as tight foreskins, a common but easily treatable problem leading to foreskin tearing—which would certainly skew the statistics.

I also find it fascinating that the male prepuce has gone straight from being an inconsequential “flap of skin” to being a complex immunological organ, just in time to be infected by a virus that targets immune cells. Is this an indication of accelerated evolution, perhaps driven by medicine's century-long obsession with the purported pathologies of male genitals, or perhaps just a demonstration of medicine's capacity to deceive the public?

It's equally fascinating that the obvious concern about the impact of male circumcision on male-to-female HIV transmission seems to be of no interest to researchers. There are good reasons to expect [ 2, 3]—and empirical evidence for (see “Heterosexual Transmission, Europe versus the United States” at http://www.circumstitions.com/HIV.html#hetero)—the thesis that male genital mutilation causes a significant increase in the rate of male-to-female HIV transmission. The net effect of circumcision in a given population may be evident in the vastly different rates of HIV infection in the United States and Europe, where routine medical genital surgery on normal, healthy, nonconsenting children is unknown. Although the collateral damage of male circumcision to women might be prevented by routine female genital mutilation, as shown in this impolitic study [ 4], one would hope common sense and decency might preemptively stop a new medical crusade against normal human anatomy.

## References

[pmed-0030071-b1] Auvert B, Taljaard D, Lagarde E, Sobngwi-Tambekou J, Sitta R (2005). Randomized, Controlled Intervention Trial of Male Circumcision for Reduction of HIV Infection Risk: The ANRS 1265 Trial. PLoS Med.

[pmed-0030071-b2] Greenhead P, Hayes P, Watts PS, Laing KG, Griffin GE (2000). Parameters of human immunodeficiency virus infection of human cervical tissue and inhibition by vaginal virucides. J Virol.

[pmed-0030071-b3] O'Hara K, O'Hara J (1999). The effect of male circumcision on the sexual enjoyment of the female partner. BJU Int.

[pmed-0030071-b4] Stallings RY, Karugendo E (2005). Female circumcision and HIV infection in Tanzania: For better or for worse? [poster] 3rd International AIDS Society Conference; 2005 24 July–27 July; Rio de Janeiro, Brazil. International AIDS Society.

